# Integrative perspectives on glycosylation networks in fungi and oomycetes

**DOI:** 10.71150/jm.2510003

**Published:** 2025-12-31

**Authors:** Heeji Moon, Hokyoung Son

**Affiliations:** 1 Department of Agricultural Biotechnology, Seoul National University, Seoul 08826, Republic of Korea; 2 Plant Health Center, Seoul National University, Seoul 08826, Republic of Korea; 3 Research Institute of Agriculture and Life Sciences, Seoul National University, Seoul 08826, Republic of Korea; 4 Plant Genomics and Breeding Institute, Seoul National University, Seoul 08826, Republic of Korea

**Keywords:** glycosylation, glycoprotein, pathogenicity, fungi, host-fungus interaction

## Abstract

Pathogenic fungi pose major threats to both global food security and human health, yet the molecular basis of their virulence remains only partially understood. Beyond genetic and transcriptional control, emerging evidence highlights protein glycosylation as a key post-translational modification that governs fungal development, stress adaptation, and host interactions. Glycosylation regulates protein folding, stability, trafficking, and immune evasion, thereby shaping infection processes across diverse pathogens. While extensively studied in model organisms, our understanding of glycosylation in pathogenic fungi remains fragmented and lacks a coherent framework linking glycosylation dynamics to fungal development and pathogenicity. This review synthesizes recent advances from proteomic, transcriptomic, and glycomic studies in pathogenic fungi, focusing on interspecific variation in glycogenes and enzymes, hierarchical regulatory networks, and glycoprotein-mediated mechanisms of virulence. Finally, we outline current challenges and highlight glycosylation-targeted strategies as promising avenues for antifungal intervention.

## Introduction

Pathogenic fungi cause devastating diseases in both plants and humans, posing severe threats to global food security and public health. These infections not only result in major agricultural losses but also impose considerable medical and social burdens (Doehlemann et al., 2017; Rokas, 2022). Accordingly, elucidating fungal pathogenic mechanisms and identifying key determinants of virulence remain central objectives in developing effective prevention and control strategies (Brown et al., 2024; Sang and Kim, 2020; Yin et al., 2023).

In recent years, research has increasingly focused on the molecular basis of fungal pathogenicity. Whereas earlier studies primarily emphasized genetic and transcriptional regulation, growing evidence highlights the pivotal roles of post-translational modifications (PTMs) in fungal development, stress adaptation, and virulence (Leach and Brown, 2012). PTMs diversify protein function through covalent modifications, including the addition of small molecules or polypeptides and site-specific chemical alterations. In eukaryotes, PTMs regulate diverse cellular processes such as apoptosis, transcription, DNA repair, cell-cycle progression, and protein–protein interactions (Sartorelli et al., 1999; Tahir et al., 2019; Zhang et al., 2004).

Among the major PTMs, glycosylation stands out for its remarkable structural complexity. Rather than functioning as a simple on/off modification, glycosylation generates a continuum of glycoforms defined by site occupancy and fine structural variation (Ives et al., 2024). Glycan assembly begins with the transfer of oligosaccharides to specific amino-acid residues—Asn for *N*-linked glycans (within the Asn-X-Ser/Thr consensus motif) and Ser/Thr for *O*-linked glycans—followed by sequential processing within the endoplasmic reticulum (ER) and Golgi apparatus. Secretory and membrane glycoproteins, after acquiring glycans through the activity of mannosyltransferases, the oligosaccharyltransferase (OST) complex, and glycosidases, are delivered to the plasma membrane, cell wall, vacuoles, lysosomes, or the extracellular space (Helenius and Aebi, 2004). These processes collectively govern protein folding, stability, trafficking, localization, abundance, and biological activity, thereby shaping fungal physiology and pathogenicity (Jiang et al., 2025; Liu et al., 2021).

The origins of glycosylation research trace back to Emil Fischer’s synthesis of simple glycosides in the 1890s, which launched the modern field of carbohydrate chemistry (Haese et al., 2022). Since then, glycosylation has been comprehensively investigated in model organisms such as humans, mice, *Arabidopsis thaliana*, and yeast (Larskaya et al., 2024; Schjoldager et al., 2020; Tanner and Lehle, 1987). In contrast, studies in fungi remain fragmented, and a unifying framework linking glycosylation to fungal development and pathogenesis is still missing. In this review, we aim to bridge this gap by systematically examining the roles of protein glycosylation in fungal pathogens. We highlight three focal areas: (1) interspecific variation in glycogenes, defined as genes encoding glycosylation enzymes, transporters, and related factors, (2) hierarchical regulatory networks of glycosylation and their impact on pathogenicity, and (3) glycosylation-mediated mechanisms of virulence. Finally, we discuss outstanding challenges and propose future research directions, emphasizing glycosylation-targeted strategies as promising avenues for antifungal intervention.

## Comparative Analysis of Glycogenes among Fungal Species

The assembly of the core glycan precursor in the ER is highly conserved across eukaryotes (Wang et al., 2017). However, subsequent modifications in the Golgi apparatus diverge markedly among lineages, reflecting the extensive genetic repertoire of enzymes that tailor glycan structures. Each fungal species harbors a distinct set of genes encoding glycosyltransferases, glucosidases, and *OST* subunits, which collectively define the diversity of both *N*- and *O*-linked oligosaccharide structures (Deshpande et al., 2008; Moon et al., 2025). Consequently, comparative analysis of glycogenes provides critical insights into species-specific glycan architectures and their functional implications for fungal biology.

### Glycosyltransferases

Glycosyltransferases are widely conserved in fungi, but their abundance and diversity vary considerably among lineages. Mannosyltransferases, for example, are far more prevalent in *Saccharomyces cerevisiae* than in filamentous fungi, reflecting its characteristic hypermannosylation. By contrast, filamentous fungi harbor a broader repertoire of genes encoding mannosyltransferases involved in high-mannose glycan maturation. This is supported by the presence of mannan polymerase I (M-Pol I; encoded by *VAN1* and *MNN9*) and M-Pol II (*MNN9*, *ANP1*, *MNN10*, *MNN11*, and *OCH1*) subunits, which occur in varying copy numbers across lineages (Moon et al., 2025).

Species-specific glycosyltransferases also give rise to distinct glycan structures. Galactosyltransferases, for instance, are uniquely present in *Magnaporthe oryzae*, *Neurospora crassa*, *Aspergillus nidulans*, and *Schizosaccharomyces pombe*, but absent in *S. cerevisiae*, *Cryptococcus neoformans*, and *Fusarium* species, consistent with the occurrence of galactose-extended glycans only in these taxa (Deshpande et al., 2008; Moon et al., 2025). Likewise, the gene *MGAT3*, encoding β-1,4-mannosyl-glycoprotein 4-*β*-N-acetylglucosaminyltransferase, is enriched in filamentous fungi, where it likely contributes to the generation of hybrid or branched *N*-glycans uncommon in yeast-form fungi (Moon et al., 2025). Another notable example is *C. neoformans*. Although the genes encoding xylose transferases have not yet been fully characterized in this species, xylose-containing glycans are consistently detected in its cell wall and capsule (Park et al., 2012; Thak et al., 2020). These unusual xylose modifications are hallmarks of *C. neoformans* glycobiology and are thought to enhance antigenicity and promote immune evasion, further underscoring how species-specific enzymatic repertoires shape distinct glycan architectures.

### Glucosidases

Glucosidases — notably α-glucosidase I and II, encoded by *GLS1* and *GLS2*, respectively — are broadly conserved, yet their proportional representation differs. Filamentous fungi maintain robust glucosidase complements, whereas *S. cerevisiae* and *S. pombe* display comparatively reduced repertoires. This correlates with the observation that in yeast, trimming is minimal and often bypassed by hypermannosylation, whereas in filamentous fungi, glucosidase-mediated processing contributes to intermediate glycan forms (e.g., Man9GlcNAc2 → Man5GlcNAc2) (De Pourcq et al., 2010). Distinct modifications further highlight functional divergence: in *Trichoderma reesei*, insufficient trimming by glucosidase II leads to abnormal glycans with phosphodiester linkages, while *Aspergillus fumigatus* produces galactofuranose (Galf)-containing *N*- and *O*-glycans, reflecting species-specific enzyme contributions to antigenic structures (De Pourcq et al., 2010).

### OST complex

Core subunits of the OST complex (Stt3, Ost1, and Wbp1) are highly conserved across fungi, reflecting their essential role in transferring Glc3Man9GlcNAc2to nascent polypeptides at the canonical *N*-glycosylation sequon Asn-X-Ser/Thr (X ≠ Pro). However, accessory subunits such as Ost3 and Ost6, required for maximal efficiency in *S. cerevisiae*, show lower sequence conservation in filamentous fungi. Moreover, homologs of Ost2, Swp1, and Ost4 are not consistently identifiable in genomes of filamentous fungi, suggesting possible functional divergence or replacement (Moon et al., 2025). These lineage-specific differences likely underlie variable occupancy of *N*-glycosylation sites and distinct glycoprotein repertoires. For example, *A. fumigatus* relies on Galf residues in its *N*-glycans and cell wall galactomannans, a structure absent in yeasts, which contributes to its immunogenicity and serves as a diagnostic marker for invasive aspergillosis (Onoue et al., 2018; Schmalhorst et al., 2008).

## Hierarchical Functional Roles of Glycosylation in Fungal Development and Pathogenesis

In recent years, substantial progress has been made in dissecting the roles of protein glycosylation in pathogenic fungi, including studies on glycogenes, structural characterization of glycans, and functional analyses of glycoproteins. However, these investigations have largely remained fragmented, focusing on isolated components rather than integrated pathways. As a result, our current understanding of how transcription factors (TFs), glycogenes, glycan structures, and glycoproteins are organized into coordinated regulatory mechanisms is still limited. A comprehensive network-level framework linking these hierarchical layers of glycosylation to fungal development and pathogenesis has yet to be established.

### TFs regulating glycogenes

TFs are central regulators that orchestrate fungal growth, development, and virulence by controlling diverse sets of downstream genes. In pathogenic fungi, large-scale TF deletion mutant libraries have been established in *Fusarium graminearum*, *C. neoformans*, and *M. oryzae*, enabling systems-level analyses of TF networks (Jung et al., 2015; Lu et al., 2014; Son et al., 2011). These studies have uncovered regulatory hubs and functional redundancies across pathways related to virulence, secondary metabolism, development, and stress adaptation. Such systems-level insights provide integrated maps that reveal crosstalk among pathways and highlight potential vulnerabilities that may serve as antifungal targets.

By contrast, only a few TFs have been directly linked to glycogenes in fungi, whereas mammalian studies provide a more detailed picture of TF–glycogene regulation. For instance, ATF2 has been described as a paradigm of multifaceted transcriptional regulation in cancer biology (Watson et al., 2017). More recently, computational frameworks have been applied to predict TFs and signaling pathways that regulate glycan biosynthesis on the genome scale (Groth et al., 2021). Members of the CREB3-like family have also emerged as central regulators, responding to nutrient availability and ER–Golgi stress and acting as transcriptional hubs coupling glycosylation with cellular homeostasis (Khan and Margulies, 2019; Sampieri et al., 2019).

To date, only a handful of fungal TFs have been implicated in glycosylation regulation, and most remain poorly characterized. As summarized in Table 1, examples include Ace2 in *Candida albicans* (Cantero and Ernst, 2011; van Wijlick et al., 2016), which regulates *O*-mannosyltransferases (*PMT*) genes; Ftg1 in *Botrytis cinerea* (Yang et al., 2024), which influences *ALG8*; and unfolded protein response regulators, represented by the functional orthologs Hac1 (*H. polymorpha*), HacA (*A. niger*), and Hxl1 (*C. neoformans*), which activate a broad spectrum of *N*-glycogenes across yeasts and filamentous fungi (Carvalho et al., 2012; Cheon et al., 2011; Glazier et al., 2015; Moon et al., 2015). In addition, stress-responsive TFs such as PacC in *Metarhizium robertsii* (Huang et al., 2015) and Ste12 in *Metarhizium acridum* (Wei et al., 2017) have been implicated in the regulation of *STT3*, *OST* genes, and *OCH1*. Despite these examples, direct DNA–TF interactions have been experimentally confirmed in only a few cases, underscoring the need for comprehensive mapping of TF–glycogene regulatory networks in pathogenic fungi.

### Glycogenes and glycan structures

**Fig. 1.**
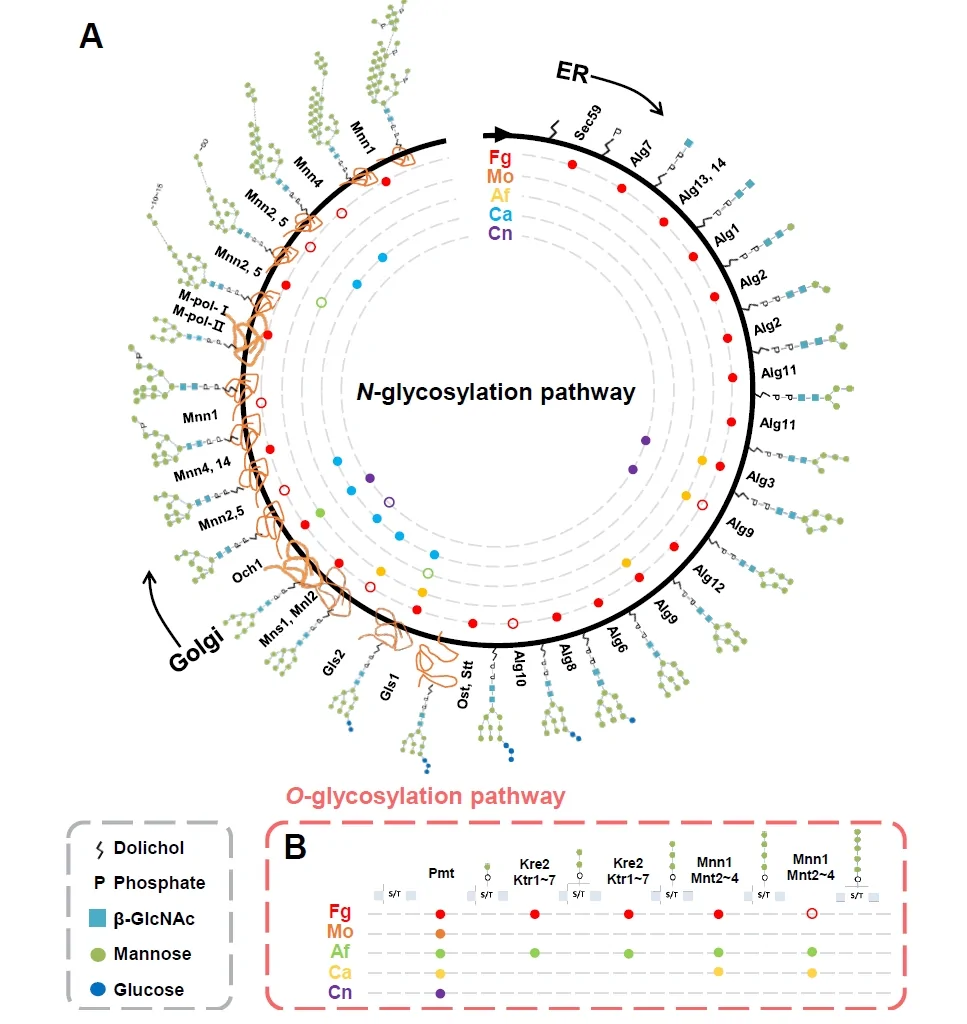
Summary of the pathogenic contributions of glycogenes associated with *N*- and *O*-glycosylation across representative fungal species. (A) Pathogenic contribution map of proteins involved in (A) *N*-glycosylation and (B) *O*-glycosylation pathways across fungal species. Species are color-coded; filled circles indicate genes with demonstrated pathogenic relevance, whereas open circles represent genes studied without pathogenic association. Abbreviations: Fg, *Fusarium graminearum*; Mo, *Magnaporthe oryzae*; Afu, *Aspergillus fumigatus*; Ca, *Candida albicans*; Cn, *Cryptococcus neoformans*. Filled circles indicate glycogenes that contribute to virulence, whereas unfilled circles represent glycogenes that have been characterized but do not affect virulence. Each positions indicate pathway step, not physical interaction.

While transcriptional regulators define the upstream control, the functional consequences of glycosylation are largely determined by the glycogenes themselves and the glycan structures they generate. Over the past decade, several components of protein glycosylation pathways have been investigated in pathogenic fungi, yet our understanding remains incomplete. Functional studies have been more extensive in human pathogens such as *A. fumigatus*, *A. nidulans*, *C. albicans*, and *C. neoformans* than in plant pathogens such as *F. graminearum* and *M. oryzae* (Fig. 1, Table 2). Notably, glycosylation research in human pathogens began earlier: in *C. albicans*, *O*- and *N*-glycogenes such as Ca*PMT*1 and Ca*MNT1* were characterized in the late 1990s, followed by Ca*OCH1*, Ca*MNN5*, and ER α-glucosidases in the mid-2000s. By contrast, functional analyses in plant pathogenic fungi began nearly a decade later, with *N*-glycogenes in *F. graminearum* first reported in 2009, and subsequent work in *F. oxysporum* and *M. oryzae* emerging mainly after 2014. This temporal gap highlights the longer history of glycosylation research in human pathogens, and its recent expansion in plant pathogens.

Comparative analyses have revealed both conserved and divergent functional roles of glycogenes. *PMT*s are universally required for cell wall integrity, morphogenesis, and virulence, but their essentiality varies. *C. albicans* Ca*PMT2* and *A. fumigatus* *PMT2* are indispensable for viability (Mouyna et al., 2010; Prill et al., 2005), whereas orthologs in *B. cinerea* or *M. oryzae* primarily affect adhesion and invasive growth (González et al., 2013, 2014; Guo et al., 2016). Similarly, *OCH1* contributes to cell wall stability across fungi, yet its role in virulence differs markedly. In *C. albicans*, the Ca*OCH1* mutant lacking the α1,6-linked polymannose backbone was attenuated in virulence (Bates et al., 2006), whereas the *OCH1* mutant in *A. fumigatus* showed no phenotypic differences from the wild type (Kotz et al., 2010). *ALG* genes, responsible for *N*-glycan precursor biosynthesis, also illustrate striking species-specific variations: complete loss of *ALG2* in *S. cerevisiae* is lethal (Giaever et al., 2002), while deletion of *MgALG2* in *Mycosphaerella graminicola* did not abolish viability but impaired cell wall integrity and morphogenesis (Motteram et al., 2011). Likewise, ER α-glucosidases (*CWH41* and *ROT2*) in human pathogens mainly affect growth and cell wall composition (Mora-Montes et al., 2007; Mota et al., 2025; Zhang et al., 2008), whereas their counterparts in *F. graminearum* also regulate secondary metabolism and sexual reproduction (Moon et al., 2025). Taken together, these comparisons highlight that although glycogenes share core functions in protein maturation and cell wall integrity, their phenotypic outputs diverge across lineages, reflecting host-specific adaptations.

Despite relatively extensive genetic studies, direct evidence linking glycogenes to their corresponding glycan structures remains limited in pathogenic fungi. As summarized in Table 2, most reports focus on phenotypic outcomes rather than directly confirming glycan products, and only a subset employed structural analyses such as high-performance liquid chromatography (HPLC), matrix-assisted laser desorption/ionization time-of-flight mass spectrometry (MALDI-TOF MS), nuclear magnetic resonance (NMR) spectroscopy, or capillary electrophoresis. For example, in *F. graminearum*, Alg12 and Alg3 were confirmed to synthesize Dol-PP-GlcNAc2Man8 and Dol-PP-GlcNAc2Man6, respectively (Moon et al., 2025). In *Fusarium oxysporum*, Galf chain biosynthesis mediated by *N*-acetylglucosaminyltransferase 2 (Gnt2) was validated through size-exclusion chromatography combined with diffusion-ordered spectroscopy (DOSY) (Lopez-Fernandez et al., 2015). Similarly, in *M. graminicola*, Alg2 was implicated in (GlcNAc)2M3 synthesis based on analyses using MALDI-TOF MS (Motteram et al., 2011), while in *A. fumigatus*, structural studies confirmed that Och1 mediates outer chain mannose addition and Galf incorporation in GalfMan6GlcNAc2 biosynthesis (Kotz et al., 2010). Despite these advances, glycomic analyses remain scarce compared with gene deletion studies, leaving many predicted glycan structures unverified.

Importantly, fungal glycans often contain unique structural features absent from mammalian systems, such as polymannose outer chains in *C. albicans* (Bates et al., 2006), Galf residues in *Aspergillus* spp. (Henry et al., 2019; Kadooka et al., 2022; Onoue et al., 2018; Schmalhorst et al., 2008), or xylose-extended mannose branches in *C. neoformans* (Thak et al., 2020). These fungal-specific glycans are integral to cell wall integrity, morphogenesis, and immune evasion, thereby representing selective vulnerabilities. Because of their structural distinctiveness and absence in the host, fungal glycan epitopes constitute promising antifungal targets.

### Glycoproteins in fungal virulence and development

Glycogenes are highly conserved across eukaryotes and are therefore less suitable as selective drug targets. In contrast, fungal glycoproteins—particularly those unique to fungal pathogens—often exhibit species-specific features that directly contribute to virulence, making them promising candidates for antifungal intervention. Similar to fungal-specific glycan structures, these glycoproteins operate at the host–pathogen interface, providing selective targets that minimize potential off-target effects in hosts.

Recent advances in glycoproteomics have enabled the large-scale identification of glycoproteins involved in cell wall integrity, signaling, and host–pathogen interactions. Most studies have focused on *F. graminearum* (Moon et al., 2025; Yu et al., 2016), *F. oxysporum* (Xu et al., 2020b), *Colletotrichum graminicola* (Mei et al., 2023), *M. oryzae* (Chen et al., 2020), *B. cinerea* (González et al., 2014), *A. nidulans* (Rubio et al., 2016), *Aspergillus niger* (Wang et al., 2012), and *C. albicans* (Léger et al., 2016). Across these diverse fungi, glycoproteomics consistently reveal common features that are critical for both fungal development and pathogenicity. Most glycoproteins are localized to the cell wall or secretory pathway, underscoring their roles at the host–pathogen interface. They typically function as adhesins, hydrolytic enzymes, or glycosylphosphatidylinositol (GPI)-anchored mannoproteins, thereby promoting adhesion, cell wall remodeling, and immune evasion (Léger et al., 2016; Rubio et al., 2016; Wang et al., 2012). Comparative analyses also demonstrate that glycoproteins rely heavily on *N*- and *O*-linked glycosylation for proper folding and stability via ER quality control (ERQC). Disruption of these processes often results in protein misfolding, defective secretion, and attenuated virulence (Chen et al., 2020; González et al., 2014; Mei et al., 2023; Moon et al., 2025). Importantly, glycoproteomic profiling has highlighted both conserved and species-specific repertoires, reflecting shared as well as specialized pathogenic strategies.

Building on these large-scale approaches, functional characterization of individual glycoproteins has further clarified their biological significance. As summarized in Table 3, these studies reveal the multifaceted roles of glycoproteins across diverse pathogenic fungi. Adhesins, such as Hwp1 and the Als family in *C. albicans* (Hoyer et al., 2008; Kamai et al., 2002; Sharkey et al., 1999; Zhao et al., 2004, 2005), and lysine motif (LysM) domain–containing proteins, including Slp1 in *M. oryzae* (Chen et al., 2014; Jones et al., 2021) and LysM (Crumière et al., 2025) in *B. cinerea*, are directly involved in host adhesion, immune evasion, and infection initiation. Secreted enzymes, such as endopolygalacturonases (Pg1 in *B. cinerea* and Pcipg2 in *Phytophthora capsici*) (González et al., 2014; ten Have et al., 1998; Sun et al., 2009), act as virulence factors by degrading host cell walls. Numerous glycoproteins also contribute to fundamental cellular processes, including vegetative growth, cell wall integrity, sporulation, and sexual reproduction, exemplified by Ecm33, Cwm1, and chitin synthases in *F. graminearum* (Fernando et al., 2019; Kim et al., 2009; Zhang et al., 2019a). ER chaperone (e.g., Cnx1) and glucosidase (e.g., Gtb1) enzymes associated with glycosylation or ERQC (Chen et al., 2020) further highlight the reliance of fungal development and pathogenicity on proper folding and trafficking. Notably, while some glycoproteins are essential for viability (e.g., phosphatases, Hsp70 family proteins) (Liu et al., 2017; Yu et al., 2014; Yun et al., 2015), others display defects only during host infection, underscoring their specialized roles in pathogenesis (Cadieux et al., 2013; Nigam et al., 2003; Pietrella et al., 2002; Son et al., 2011; Yin et al., 2018a; Yun et al., 2015). Together, these studies illustrate how glycoproteins function at the interface of fungal physiology and pathogenicity, reinforcing their significance as selective and promising targets for antifungal intervention.

## Potential Regulatory Mechanisms of Glycosylation in Fungal Virulence

**Fig. 2.**
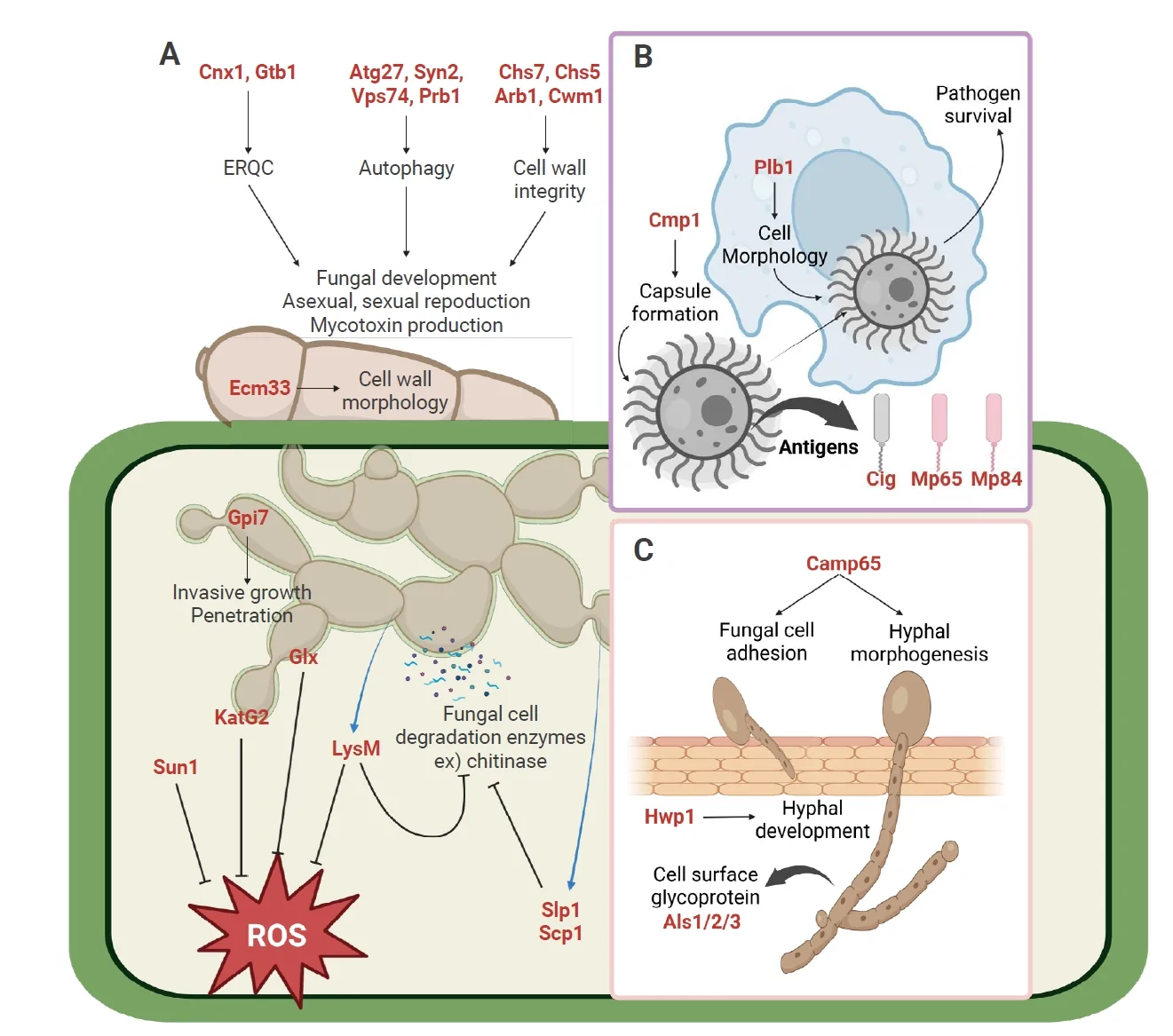
Potential regulatory mechanisms of glycoproteins in fungal virulence. Functional analyses of glycogenes have been conducted in both human and plant pathogenic fungi. Conserved glycoproteins contribute to key processes such as protein maturation, cell wall integrity, morphogenesis, and virulence, yet their functional impacts vary in a species-specific manner across fungal lineages. (A) In plant pathogenic fungi, glycosylation regulates penetration and invasive growth by stabilizing and positioning key glycoproteins required for invasive growth, cell wall remodeling, immune evasion, and oxidative stress tolerance. These proteins can be grouped into ER quality control (ERQC; Cnx1, Gtb1, and Ecm33), autophagy and vesicle trafficking (Atg27, Syn2, Vps74, and Prb1), and cell wall integrity factors (Cwm1, Chs7, Chs5, and Arb1), all contributing to fungal development, sporulation, and mycotoxin production. Additional cell wall–localized or secretory proteins, such as Gpi7, KatG2, Glx, and LysM effectors (LysM1, Slp1, Scp1), contribute to invasive growth by scavenging host-derived reactive oxygen species and protecting fungal cell walls from plant chitinases. (B) In the human pathogen *C. neoformans*, several glycoproteins function as antigens and virulence determinants at the cell surface. Cig1, Mp65, and Mp84 act as immunogenic proteins that mediate host–pathogen interactions, while Cmp1 regulates capsule formation and Plb1 contributes to intracellular infection and morphological adaptation. Capsule polysaccharides further support immune evasion and pathogen survival. (C) In *C. albicans*, glycoproteins orchestrate adhesion, morphogenesis, and hyphal development. MP65 promotes fungal adhesion and morphogenetic transitions; Hwp1 facilitates hyphal development and stable adhesion to host tissues; and the Als family proteins (Als1, Als2, and Als3) function as major surface adhesins driving biofilm formation and immune evasion. This figure was created using BioRender.

While a comprehensive, network-level framework linking the hierarchical layers of glycosylation has yet to be fully established, emerging evidence suggests functional interconnections across these levels. Here, we outline glycoprotein-centered virulence networks (Fig. 2), proposing that glycosylation governs fungal pathogenicity through a multilayered system integrating ERQC, secretory trafficking, cell wall biogenesis, and the activity of surface and secreted glycoproteins at the host interface. To illustrate these concepts, we highlight two representative contexts: plant pathogenic fungi and human pathogenic fungi, focusing on *C. neoformans* and *C. albicans*.

### Plant pathogenic fungi

In plant pathogens, glycosylation underpins penetration and invasive growth by stabilizing and localizing key glycoproteins required for appressorium formation, cell wall remodeling, immune evasion, and oxidative stress tolerance. Functionally, these glycoproteins can be broadly grouped into five categories: ERQC, autophagy, cell wall integrity, reactive oxygen species (ROS) resistance, and defense against host-derived chitinases and other antifungal enzymes (Fig. 2A).

The ERQC system is indispensable for proper protein folding and stability, thereby shaping fungal virulence. In *M. oryzae*, Cnx1 and Gtb1 are required for vegetative growth, conidiation, and invasive hyphal growth (Chen et al., 2020). In *C. graminicola*, the ERQC components such as Cnx1 likewise ensure effector stability and secretion, underscoring their central role in host–pathogen interactions (Mei et al., 2023).

Vesicle trafficking and autophagy are tightly interconnected, coordinating fungal development, stress adaptation, and secondary metabolism. In *F. graminearum*, the SNARE protein Syn2 is required for sexual development (Hong et al., 2010), while the vacuolar sorting factor Vps74 contributes to vegetative growth, conidiation, sexual reproduction, and mycotoxin production (Kim et al., 2015b). Autophagy-related factors intersect with pathogenic processes: Atg27 mediates selective autophagy pathways such as mitophagy, pexophagy, and the cytoplasm-to-vacuole targeting (Cvt) pathway, influencing development and mycotoxin production (Lv et al., 2017). Similarly, the subtilisin-like protease Prb1 contributes to mycotoxin biosynthesis, lipid metabolism, and environmental stress responses, further linking autophagy to virulence (Xu et al., 2020a).

Cell wall integrity is fundamental for fungal survival and virulence, serving as a dynamic barrier that both protects against stresses and mediates host–pathogen interactions. In *F. graminearum*, chitin synthases Chs7 and Chs5 are required for growth, septum formation, and perithecium development (Kim et al., 2009). Arb1, an ATP-binding protein, plays a broader role by contributing to infective growth, oxidative stress resistance, cell wall maintenance, mycotoxin production, and sexual/asexual development (Yin et al., 2018a). Cwm1, a cell wall mannoprotein, is essential for vegetative growth and the cell wall integrity (Zhang et al., 2019a). In *M. oryzae* (Liu et al., 2020), a GPI-anchored protein Gpi7 drives penetration, invasive growth, and cell wall biogenesis.

Secretory and cell wall-associated proteins act directly at the pathogen-host interface. In *F. graminearum*, KatG2, a wall-localized peroxidase, detoxifies ROS during invasion (Guo et al., 2019), while Glx, a plasma-membrane glyoxal oxidase, contributes to both ROS detoxification and to mycotoxin production (Song et al., 2016). Ecm33, a GPI-anchored protein, is required for maintaining proper cell wall morphology and integrity (Fernando et al., 2019). Multiple LysM effectors act as secretory proteins that counteract host defense enzymes: Lysm1 in *B. cinerea*, Slp1 in *M. oryzae*, and Scp1 in *Lasiodiplodia theobromae* (Chen et al., 2014; Crumière et al., 2025; Jones et al., 2021; Peng et al., 2022). Notably, Lysm1 in *B. cinerea* also scavenges ROS, underscoring its dual protective role.

### Human pathogenic fungi

In *C. neoformans*, cell surface glycoproteins such as Cig1, Mp65, and Mp84 (Fig. 2B) act as key antigens that facilitate adhesion, modulate immune responses, and promote persistence (Cadieux et al., 2013; Pietrella et al., 2002; Teixeira et al., 2014). Their antigenic properties enhance host recognition while simultaneously enabling immune evasion. Capsule formation is also a major virulence determinant: the polysaccharide capsule, composed primarily of glucuronoxylomannan (GXM)- and galactoxylomannan (GalXM)-rich capsule blocks immune recognition, inhibits phagocytosis, and modulates cytokine signaling. Cmp1 regulates capsule biosynthesis (Han et al., 2020), while Plb1 facilitates intracellular infection and alters fungal cell morphology during infection (Evans et al., 2015; Thak et al., 2020). Taken together, these glycoproteins and capsule-associated factors orchestrate the multifaceted virulence program of *C. neoformans*.

In *C. albicans*, several glycoproteins play pivotal roles in virulence by orchestrating adhesion, morphogenesis, and hyphal development (Fig. 2C). MP65 mediates fungal cell adhesion and hyphal morphogenesis, thereby promoting initial host colonization and the morphological transitions essential for invasive growth (Sandini et al., 2007). Hwp1, a hypha-specific glycoprotein, supports hyphal development and acts as a substrate for host transglutaminases, enhancing stable adhesion to epithelial surfaces (Sharkey et al., 1999; Staab et al., 2013). The Als family proteins, particularly Als1, Als2, and Als3, are major cell surface adhesins that mediate binding to host tissues and abiotic substrates, facilitating biofilm formation and immune evasion (Hoyer et al., 2008; Kamai et al., 2002; Zhao et al., 2004). Collectively, these glycoproteins underscore the multifactorial nature of *C. albicans* pathogenicity, linking surface adhesion and morphogenetic plasticity to host invasion and persistence.

## Future Perspectives and Conclusion

Over the past decade, numerous studies have highlighted the fundamental roles of glycosylation in fungal infection. It is now evident that fungal glycosylation is intricately involved in diverse aspects of pathogenicity, including growth, morphogenesis, cell wall biogenesis, protein secretion, and host immune modulation. Future research should therefore move beyond gene-centric approaches toward a more integrated understanding of how glycosylation drives fungal virulence. Recent advances integrating proteomics, transcriptomics, and glycomics in species such as *F. graminearum*, *M. oryzae*, *C. graminicola*, and *C. neoformans* have begun to unravel the multilayered roles of glycosylation in fungal virulence (Chen et al., 2020; Mei et al., 2023; Moon et al., 2025; Mota et al., 2025). Nevertheless, an overarching framework that links the hierarchical layers of glycosylation with fungal development and pathogenesis has yet to be established. In this review, we summarize the current knowledge and emphasize the need for such integrative models.

Despite these advances, significant challenges remain. The first major challenge is the comprehensive profiling of glycan structures in pathogenic fungi. While analogous efforts in bacteria, viruses, and cancer research have progressed rapidly, fungal systems remain relatively underexplored. For instance, in SARS-CoV-2, the high-mannose and complex glycan shield of the spike protein has been targeted using glycan-binding lectins and antibodies (Klevanski et al., 2024). In bacteria, the *O*-antigen glycans of Gram-negative species have emerged as promising vaccine and antibody targets (Zhang et al., 2009). In cancer, tumor-associated carbohydrate antigens (TACAs) such as sialylated epitopes and aberrant *N*-glycan branching are under active investigation as therapeutic targets (Rashidijahanabad and Huang, 2020; Zhou et al., 2025). In contrast, antifungal strategies aimed at glycan structures remain at an early stage. Although resources such as the GlycoShape database provide approximations of glycoprotein structures (Ngolong Ngea et al., 2021), the development of clinically relevant antifungal targets will require precise and systematic profiling of pathogen-specific glycans. Recent studies have begun to characterize glycan landscapes in *C. neoformans*, *F. graminearum*, and *A. fumigatus* (Moon et al., 2025; Onoue et al., 2018; Park et al., 2012). Moving forward, these efforts must expand to include comparative profiling of host glycomes in plants and animals. Such integrative analyses will help identify pathogen-specific glycan motifs, thereby providing a foundation for the rational design of antifungal agents.

The second challenge is to elucidate whether and how distinct glycan structures directly influence the functions of their cognate glycoproteins. Large-scale glycoproteomic studies have identified numerous glycoproteins associated with virulence (Chen et al., 2020; Mei et al., 2023; Moon et al., 2025; Yu et al., 2016), but a critical next step is to determine whether their functions depend simply on the presence of glycans, or on their specific structural features. The absence of glycosylation at a given site can be examined through site-directed mutagenesis of the corresponding amino acid residues (Guo et al., 2019; Mei et al., 2023; Peng et al., 2022). In contrast, assessing the contribution of glycan structure requires more advanced approaches, including LC–MS/MS of released glycans or glycopeptides, exoglycosidase digestion, and complementary techniques such as NMR or lectin-based profiling. Furthermore, experimental systems such as glycoengineering of fungal pathways, application of glycosidase inhibitors, and chemoenzymatic remodeling of glycoproteins can generate distinct glycoforms of the same protein. By comparing the activities, stability, and virulence-related properties of these glycoforms, researchers can directly evaluate the functional relevance of glycan structural variation. Such analyses will be essential for establishing whether particular glycan motifs represent viable and selective targets for antifungal drug development.

## Figures and Tables

**Table 1.** TFs regulating glycogenes

Species	Gene	Type	Description	Regulated genes	Direct binding	Reference
*C. albicans*	*CaACE2*	Zinc-finger	RAM (regulation of *ACE2* TF and polarized morphogenesis) signaling network	*PMT*s	Not studied	Cantero and Ernst (2011); van Wijlick et al. (2016)
*B. cinerea*	*BcFTG1*	Zinc-finger	fungal TF containing the GAL4 domain	*ALG8*	Not studied	Yang et al. (2024)
*H. polymorpha*	Hp*HAC1*	bZIP	Major unfolded protein response (UPR)	*ALG5, MNN2, MNN4, KTR1, OST1,OST2, OST4, OCR5, SWP1, WBP1*	Not studied	Moon et al. (2015)
*A. niger*	*hacA*	bZIP	Major unfolded protein response (UPR)	*ALG6, ALG8, ALG2, ALG3, DPM1,DPM2, ALG5, ALG7, RFT1, ALG9, SEC59, ALG12, ALG10, OST1, WBP1,OST2, OST3, SST3*	Not studied	Carvalho et al. (2012)
*C. neoformans*	*HXL1*	bZIP	*HAC1*, XBP1-like gene 1	*UPR* genes (*WBP1, PMT1, PMT2, PMT4, WBP1, ALG7, OST1*)	Not studied	Cheon et al. (2011); Glazier et al. (2015)
			Mediate the unfolded protein response (UPR) pathway			
*M. robertsii*	*MrpacC*	C2H2-type zinc-finger	pH-responsive TF	*STT3*, *OST3*, * PMT4*	*STT3, OST3, PMT4*	Huang et al. (2015)
*M. acridum*	*MaSte12*	C2H2-type zinc-finger	Direct target and functions downstream of the mitogen-activated protein kinase (MAPK) Fus3/Kss1	*OCH1*	Not studied	Wei et al. (2017)

**Table 2.** *N&O*-Glycogenes and glycan structures discussed in this review

Species	Gene	Virulence (O = affected; X = no effect)	Biological function(s)	Glycan structure	Reference
*B. cinerea*	*bcpmt1*	O	Morphogenesis	Not studied	González et al. (2013, 2014)
			Fungal adherence		
			Cell wall integrity		
	*bcpmt2*	O	Morphogenesis	Not studied	González et al. (2013, 2014)
			Fungal adherence		
			Cell wall integrity		
	*bcpmt4*	O	Morphogenesis	Not studied	González et al. (2013, 2014); Plaza et al. (2025)
			Fungal adherence		
			Cell wall integrity		
*C. graminicola*	*Cgalg3*	O	ER homeostasis	Not studied	Mei et al. (2023)
			Fungal growth		
*F. graminearum*	*RFT1*	O	Mycotoxin synthesis	Not studied	Moon et al. (2025)
			Vegetative growth		
			Conidiation		
			Sexual development		
	*CWH41 (MOGS)*	X	Mycotoxin synthesis	Not studied	
			Conidiation		
			Sexual development		
	*ROT2 (GLS2, GANAB)*	X	6 orthologs showed different phenotypes	Not studied	
			Mycotoxin synthesis		
			Vegetative growth		
			Conidiation		
			Sexual development		
	*ALG5*	O	Vegetative growth	Not studied	
			Sexual development		
	*ALG6*	O	Vegetative growth	Not studied	
			Sexual development		
	*ALG8*	O	Mycotoxin synthesis	Not studied	
			Vegetative growth		
			Sexual development		
	*PSA1 (MPG)*	O	Mycotoxin synthesis	Not studied	
			Vegetative growth		
			Conidiation		
			Sexual development		
	*MNS1 (MAN1B)*	O	2 orthologs showed different phenotypes	Not studied	
			Mycotoxin synthesis		
			Conidiation		
	*MNL2 (MAN1A)*	O	4 orthologs showed different phenotypes	Not studied	
			Mycotoxin synthesis		
			Vegetative growth		
			Conidiation		
	*PMT1, 5, 7*	O	Vegetative growth	Not studied	
			Conidiation		
			Sexual development		
	*MNN14*	X	Mycotoxin synthesis	Not studied	
			Vegetative growth		
			Conidiation		
	*ALG1*	X	Vegetative growth	Not studied	
	*ALG11*	O	Mycotoxin synthesis	Not studied	
			Vegetative growth		
			Conidiation		
			Sexual development		
	*ALG12*	O	Vegetative growth	Biosynthesis of the Dol-PP-GlcNAc2Man8 (HPLC)	
			Sexual development		
	*ALG2*	o	Mycotoxin synthesis	Not studied	
			Vegetative growth		
			Conidiation		
			Sexual development		
	*ALG3*	O	Mycotoxin synthesis	Biosynthesis of the Dol-PP-GlcNAc2Man6 (HPLC)	
			Vegetative growth		
			Conidiation		
			Sexual development		
	*ALG9*	X	Mycotoxin synthesis	Not studied	
			Vegetative growth		
	*HOC1*	O	Mycotoxin synthesis	Not studied	
	*OCH1*	O	Mycotoxin synthesis	Not studied	
			Vegetative growth		
			Conidiation		
	*KTR5, KTR7*	O	Mycotoxin synthesis	Not studied	
			Vegetative growth		
			Conidiation		
			Sexual development		
	*KTR4*	O	Vegetative growth	Not studied	
			Conidiation		
	*KRE2, KTR1, 2, 3, 6*	X	Mycotoxin synthesis	Not studied	
			Vegetative growth		
			Sexual development		
	*MNT4*	X	Mycotoxin synthesis	Not studied	
			Conidiation		
	*MNN1, MNT3*	X	Mycotoxin synthesis	Not studied	
	*MNN10*	X	Conidiation	Not studied	
			Sexual development		
	*MNN11*	X	Mycotoxin synthesis	Not studied	
			Vegetative growth		
			Conidiation		
			Sexual development		
	*MNN2, 5*	X	Conidiation	Not studied	
	*MNN4*	O	Mycotoxin synthesis	Not studied	
			Vegetative growth		
	*VAN1*	X	Mycotoxin synthesis	Not studied	
			Vegetative growth		
			Sexual development		
	*ANP1*	O	Mycotoxin synthesis	Not studied	
			Vegetative growth		
			Conidiation		
			Sexual development		
	*MNN9*	O	Vegetative growth	Not studied	
			Sexual development		
	*STT3*	O	Not changed	Not studied	
	*SWP1*	X	Conidiation	Not studied	
	*WBP1*	O	Mycotoxin synthesis	Not studied	
			Vegetative growth		
			Conidiation		
*F. oxysporum*	*OCH1*	O	Fungal growth	Not studied	Li et al. (2014)
			Cell wall integrity		
			hyphal adhesion		
	*GNT2*	O	Conidium size and morphology	Biosynthesis of the Galactofuranose chains	Lopez-Fernandez et al. (2015)
			Oxidative stress resistance	(size-exclusion chromatography with Diffusion Ordered Spectroscopy (DOSY))	
			cell wall organization, biogenesis and remodelling hyphal fusion		
			Secretion of trafficking vesicles		
*F. oxysporum *f. sp.* cucumerinum*	*PMT1*	O	Fungal growth	Not studied	Li et al. (2014)
			Cell wall integrity		
			Conidiation		
	*PMT2*	O	Fungal growth	Not studied	Li et al. (2014)
			Cell wall integrity		
			Conidiation		
	*PMT4*	O	Fungal growth	Not studied	Xu et al. (2020b)
			Cell wall integrity		
			Conidiation		
*M. graminicola*	*MgAlg2*	O	Cell wall integrity	Biosynthesis of the (GlcNAc)2M3	Motteram et al. (2011)
			Yeast-like to filamentous growth switch	(MALDI-ToF-MS)	
*M. oryzae*	*GLS1*	O	Mycelial growth	Not studied	Chen et al. (2020)
			Conidiation		
			Invasive hyphal growth		
	*GLS2*	O	Mycelial growth	Not studied	Chen et al. (2020)
			Conidiation		
			Invasive hyphal growth		
	*GTB1*	O	Mycelial growth	Not studied	Chen et al. (2020)
			Conidiation		
			Invasive hyphal growth		
	*ALG3*	O	Mycelial growth	Not studied	Chen et al. (2014)
			Conidiation		
			Invasive growth		
			Oxidative stress resistance		
	*PMT2*	O	Fungal adhesion	Not studied	Guo et al. (2016)
			Conidial germination		
			cell wall integrity		
			Invasive hyphae growth		
	*PMT4*	O	Hyphal growth	Not studied	Pan et al. (2019)
			Conidiation		
			Penetration and biotrophic invasion		
	*ALG9*	O	Conidial production	Not studied	Zhang et al. (2025)
			Appressorium formation		
			Responses to stressors		
*P. capsici*	*PcSTT3B*	O	Vegetative growth	Not studied	Cui et al. (2023)
			Sporangial release rate		
			Zoospore production		
*P. digitatum*	*pdpmt2*	O	Cell wall integrity	Not studied	Harries et al. (2015)
			Conidiogenesis		
			Sensitivity to fungicide		
*U. maydis*	*gls1*	O	Initial stages of biotrophic growth	Not studied	Fernández-Álvarez et al. (2013)
	*gas1*	O	Intracellular expending	Not studied	Schirawski et al. (2005)
	*gas2*	O	Intracellular expending	Not studied	Fernández-Álvarez et al. (2013)
	*pmt4*	O	Appressorium formation	Not studied	Fernández-Álvarez et al. (2009)
			Plant cuticle penetration		
*V. dahliae*	*VdOCH1*	O	Hyphal growth	Not studied	Zhang et al. (2019b)
			Conidia production		
			Microsclerotia formation		
			Cell wall integrity		
	*VdSTT3*	O	Fungal development	Not studied	Su et al. (2018)
			Hyphal growth		
			Glycoprotein secretion		
*A. fumigatus*	*CWH41*	X	Cell wall integrity (CWI)	Not studied	Zhang et al. (2008)
			Polarity		
			Septation		
			Conidiation		
	*AMS1*	Not studied	Conidial formation	Not studied	Li et al. (2009)
			Polarity		
			Septation		
	*OCH1*	O	Sporulation	Biosynthesis of the Outer glycan chain	Kotz et al. (2010)
				(Capillary electrophoresis DNA Sequencer)	
	*PMT1*	X	No discernible phenotype	Not studied	Mouyna et al. (2010)
			Δ*PMT1/PMT4* was lethal		
	*PMT2*	O	Essential	Not studied	Mouyna et al. (2010)
	*PMT4*	X	Mycelial growth	Not studied	Mouyna et al. (2010)
			Conidiation		
			Sensitivity to echinocandins		
			Δ*PMT1/PMT4* was lethal		
	*ANPA*	X	Mycelial growth	Core-mannan structure of fungal-type galactomannan (FTGM)	Kadooka et al. (2022)
			Conidia formation	(1H-NMR spectroscopy)	
	*MNN9*	X	No discernible phenotype	Not involved in the synthesis of the FTGM α-core-mannan	Du et al. (2019); Kadooka et al. (2022)
			Apical growth and polarity	(1H-NMR spectroscopy)	
				No significant role in the biosynthesis of N-glycans	
				(MALDI-TOF MS)	
	*VAN1*	X	No discernible phenotype	Not involved in the synthesis of the FTGM α-core-mannan	Kadooka et al. (2022)
				(1H-NMR spectroscopy)	
	*KTR4*	O	Polarized growth	Polymerization galactomannan	Henry et al. (2019)
			Conidiation	(HPLC analysis)	
			Conidial viability		
	*KTR7*	O	Polarized growth	Polymerization galactomannan	Henry et al. (2019)
			Conidiation	(HPLC analysis)	
			Conidial viability		
	*CMSA*	Not studied	Mycelial growth	Biosynthesis of the FTGM core-mannan structure	Onoue et al. (2018)
			sensitive to antifungal agents	(Proton nuclear magnetic resonance (1H-NMR) spectroscopy)	
	*CMSB*	Not studied	Mycelial growth	Biosynthesis of the FTGM core-mannan structure	Onoue et al. (2018)
			sensitive to antifungal agents	(Proton nuclear magnetic resonance (1H-NMR) spectroscopy)	
	*MNT1*	O	Cell wall stability	Not studied	Wagener et al. (2008)
	*MNN2*	Not studied	No discernible phenotype	*ΔMNN2/MNN5*	Kadooka et al. (2024)
			Δ*MNN2/MNN5* exhibited a growth defect and abnormal conidial formations	Biosynthesis of the outer chain like α-(1→6)-linked mannan	
				(1H-NMR analysis)	
	*MNN5*	Not studied	No discernible phenotype	*ΔMNN2/MNN5*	Kadooka et al. (2024)
			Δ*MNN2/MNN5* exhibited a growth defect and abnormal conidial formations	Biosynthesis of the outer chain like α-(1→6)-linked mannan	
				(1H-NMR analysis)	
	*STT3*	Not studied	Fungal growth	Not studied	Li et al. (2011)
			Cell wall integrity (CWI)		
	*GALF*	O	Morphology and growth	Biosynthesis of the GalfMan6GlcNAc2	Schmalhorst et al. (2008)
			Susceptible to drugs	(Four-capillary electrophoresis DNA sequencer)	
*A. nidulans*	*ALG7*	Not studied	Failed to delete	Not studied	Gerhardt et al. (2025)
	*ALG6 (ALGF)*	Not studied	Not studied	Not studied	Anyaogu et al. (2021); Gerhardt et al. (2025)
	*AMS1*	X	No visible effect on growth or morphology	Not studied	Anyaogu et al. (2021); Eades et al. (1998)
			Recycling macromolecular components		
	*AN5748*	Not studied	Fungal growth	Not studied	Gerhardt et al. (2025)
			Sporulation		
			Resistance to sorbitol and tunicamycin		
	*PMTA*	Not studied	Fungal growth	Not studied	Oka et al. (2004)
			Cell wall formation		
			*ΔPMTA/PMTC* lethal		
	*PMTB*	Not studied	Conidiation	Not studied	Goto et al. (2009)
			Polarity maintenance		
			Δ*PMTB/PMTC* lethal		
	*PMTC*	Not studied	Fungal growth	Not studied	Goto et al. (2009)
			Cell wall integrity		
			Osmotic stabilization		
			Δ*PMTA/PMTC*, Δ*PMTB/PMTC* lethal		
	*ALG1*	Not studied	Failed to delete	Not studied	Gerhardt et al. (2025)
	*ALG2*	Not studied	Fungal growth	Not studied	Gerhardt et al. (2025)
			Sporulation		
	*ALG11*	Not studied	Failed to delete	Not studied	Gerhardt et al. (2025)
	*ALG3 (ALGC)*	Not studied	Sensitive to tunicamycin and calcium stress	Biosynthesis of the Man5-7GlcNAc2	Anyaogu et al. (2021); Gerhardt et al. (2025)
				(UHPLC-FLR-MS)	
	*ALG9 (ALGI)*	Not studied	Sensitive to tunicamycin and calcium stress	Not studied	Anyaogu et al. (2021); Gerhardt et al. (2025)
	*ALG12 (ALGL)*	Not studied	Failed to delete	Not studied	Anyaogu et al. (2021); Gerhardt et al. (2025)
	*ALG13*	Not studied	Failed to delete	Not studied	Gerhardt et al. (2025)
*C. albicans*	*CaCWH41*	O	Growth rates	Not studied	Mora-Montes et al. (2007)
			Cell wall composition		
	*CaROT2*	o	Growth rates	Not studied	Mora-Montes et al. (2007)
			Cell wall composition		
	*CaMNS1*	O	Growth rates	Not studied	Mora-Montes et al. (2007)
			Cell wall composition		
	*CaPMT2*	O	Essential	Not studied	Prill et al. (2005)
	*CaPMT4*	O	Fungal growth	Not studied	Prill et al. (2005)
			Morphogenesis		
			Antifungal resistance.		
	*CaPMT5*	X	Fungal growth	Not studied	Prill et al. (2005)
			Morphogenesis		
			Antifungal resistance.		
	*CaPMT1*	O (heterozygous)/	Hyphal morphogenesis	Not studied	Eades et al. (1998); Timpel et al. (2000)
		X (homozygous)	Supersensitivity to the antifungal agents		
	*CaPMT6*	O	Morphogenesis	Not studied	Timpel et al. (2000)
			Antifungal sensitivities		
	*CaOCH1*	O	Temperature-sensitive growth	Biosynthesis of the N-glycan outer chain	Bates et al. (2006)
			Cellular aggregation	(ES-MS analysis, Gas chromatography-mass spectrometry)	
			Host-fungal interaction		
	*CaMNT1*	O	Adhesion	Biosynthesis of the second mannose to O-glycan	Buurman et al. (1998)
				(Biogel-P4 chromatography)	
	*CaMNN5*	O	Hyphal growth	Biosynthesis of the N-linked mannan branches	Bai et al. (2006)
			Iron homeostasis	(Thin-layer chromatography and TLC plate to autoradiography)	
			Cell wall integrity		
			Morphogenesis		
	*CaMNT4*	Not studied	No discernible phenotype	Not studied	Mora-Montes et al. (2010)
			ΔMNT3/MNT4/MNT5 exhibited severe growth defect		
			ΔMNT4/MNT5, ΔMNT3/MNT4/MNT5 altered cell wall composition		
	*CaMNT5*	O (Δ*MNT3/MNT5*)	No discernible phenotype	Not studied	Mora-Montes et al. (2010)
			Δ*MNT3/MNT4/MNT5* exhibited severe growth defect		
			Δ*MNT4/MNT5*, Δ*MNT3/MNT4/MNT5* altered cell wall composition		
	*CaMNT3*	O (Δ*MNT3/MNT5*)	No discernible phenotype	Not studied	Mora-Montes et al. (2010)
			Δ*MNT3/MNT5*, Δ*MNT3/MNT4/MNT5* exhibited severe growth defect		
			Δ*MNT3/MNT5*, Δ*MNT4/MNT5*, Δ*MNT3/MNT4/MNT5* altered cell wall composition		
*C. neoformans*	*MNS1*	(Δ*MNS1/MNS101*)	Sensitive to ER stress (tunicamycin, DTT)	Remove mannose from GlcNAc2Man9 to GlcNAc2Man8	Mota et al. (2025)
			Sensitive to ER stress cell wall integrity stress	(HPLC and MALDI-TOF)	
			Δ*MNS1/MNS101* growth defect		
	*MNS101*	(Δ*MNS1/MNS101)*	Sensitive to ER stress (tunicamycin, DTT)	Remove mannose from (> M10) glycan or M8 further trimming	Mota et al. (2025)
			Sensitive to ER stress cell wall integrity stress	(HPLC and MALDI-TOF)	
			Δ*MNS1/MNS101* growth defect		
	*MNL1*	X	No discernible phenotype	Involved in targeting misfolded proteins for ERAD	Mota et al. (2025)
				rather than in normal N-glycan processing	
				(HPLC, MALDI-TOF)	
	*MNL2*	X	No discernible phenotype	Involved in targeting misfolded proteins for ERAD	Mota et al. (2025)
				rather than in normal N-glycan processing	
				(HPLC, MALDI-TOF)	
	*PMT4*	O	Fungal growth	Not studied	Olson et al. (2007); Willger et al. (2009)
			Cell wall integrity		
			Cell morphology		
	*PMT2*	O	Essential	Not studied	Willger et al. (2009)
	*PMT1*	O	Cell morphology and integrity	Not studied	Willger et al. (2009)
			ΔPMT1/PMT4 is synthetically lethal,		
	*ALG3*	O	Macrophage cell death	Biosynthesis of the Dol-PP-GlcNAc2Man6	Thak et al. (2020)
				(HPLC, MALDI-TOF)	
	*OCH1*	Slightly attenuated,	No discernible phenotype	Addition of a single α1,6-linked mannose residue to the Man8GlcNAc2 core	Park et al. (2012)
		not critical		(HPLC, MALDI-TOF)	
	*ALG9*	O	Sensitive to SDS, fluconazole	Biosynthesis of the Dol-PP-GlcNAc2Man7	Thak et al. (2020)
				(HPLC, MALDI-TOF)	
	*MNN2*	Not studied	No discernible phenotype	Biosynthesis of the elongated outer chain N-glycan	Park et al. (2012)
				(HPLC, MALDI-TOF)	
	*KTR3*	Not studied	Cell wall stability	Not involved in the processing of N-glycans (M6–10, X1M6–10)	Park et al. (2012)
				(HPLC, MALDI-TOF)	

Man9GlcNAc2 : Glycan compositions with 9 mannose and two N-acetylglucosamine residues. X1M6–10: Extended high-mannose glycans containing one Xylose and 6–10 mannose residues. FTGM: Fungal-type galactomannan.

**Table 3.** Glycoproteins in fungal virulence and development

Species	Gene	Description	Phenotypic defect(s)	Reference
*B. cinerea*	*bcsun1*	Member of the β-glucosidase SUN family	Fungal morphogenesis	Pérez-Hernández et al. (2017)
		(secreted glycoprotein)	Cell Wall Integrity	
			Reproductive structures formation	
			ROS sensitivity	
	*bcpg1*	Endopolygalacturonase	Studied only for virulence	González et al. (2014); ten Have et al. (1998)
	*bclysm1*	Lysin motif domains	Protects hyphae against degradation by external chitinases	Crumière et al. (2025)
			Prevent them from inducing ROS	
			Infection initiation	
			Adhesion to host	
*C. graminicola*	*Cgcnx1*	ER chaperone	(Site directed mutagenesis)	Mei et al. (2023)
			Vegetative growth	
			Effector stability and secretion	
*F. graminearum*	*CFEMN1*	Contain a CFEM domain	Interact with extracellular binding	Zuo et al. (2022)
			proteins from host	
	*TUB2*	β2-tubulin	Vegetative growth	Liu et al. (2013);
			Sensitive to carbendazim	Zhao et al. (2014)
	*ADE5*	Phosphoribosylamine-glycine ligase	Adenine auxotrophy	Kim et al. (2007)
			Vegetative growth	
			Sexual reproduction	
	*CHS7*	Chitin synthase	Vegetative growth	Kim et al. (2009)
			Septa formation	
			Perithecia formation	
	*CHS5*	Chitin synthase	Vegetative growth	Kim et al. (2009)
			Septa formation	
			Perithecia formation	
	*VPS74*	Vacuolar protein sorting	Vegetative growth	Kim et al. (2015b)
			Conidiation	
			Sexual development	
			Mycotoxin production	
	*SYN2*	Syntaxin-like SNARE genes	Self and female fertility	Hong et al. (2010)
			Localized in plasma membranes and septa	
	*ERG9*	Ergosterol biosynthesis	Essential	Yun et al. (2014)
	*PRB1*	Subtilisin-like protease belonging to proteinase K-like subfamily	Mycotoxin production	Xu et al. (2020a)
			Responses to environmental stimuli	
			Lipid metabolism	
			Autophagy regulation	
	*ATG27*	Autophagy-related genes	Vegetative growth	Lv et al. (2017)
			Sporulation	
			Mycotoxin production	
			Autophagy regulation	
	*ILV6*	Acetohydroxyacid synthase	BCAA-auxotroph	Liu et al. (2015)
			Vegetative growth	
			Pigmentation	
			Mycotoxin production	
	*ILV2*	Acetohydroxyacid synthase	BCAA-auxotroph	Liu et al. (2015)
			Vegetative growth	
			Conidial formation	
			Pigmentation	
			Mycotoxin production	
	*GPB1*	G protein	Mycotoxin production	Yu et al. (2008)
			Chitin accumulation	
	*RAB11*	Ras-related proteins in brain	Polarized growth and/or exocytosis	Zheng et al. (2015)
	*KAR*	Hsp70s	Essential	Liu et al. (2017)
	*SSC*	Hsp70s	Essential	Liu et al. (2017)
	*SSB*	Hsp70s	Proper folding of β2-tubulin	Liu et al. (2017)
			Vegetative growth	
			Low temperature response	
			Vacuole fusion and endocytosis	
			Mycotoxin production	
	*SSA*	Hsp70s	Essential	Liu et al. (2017)
	*CAP1*	Cyclase-associated protein	Vegetative growth	Yin et al. (2018b)
			Conidiogenesis	
			Mycotoxin production	
	*KATG2 /FCA7*	peroxidase	(Site directed mutagenesis)	Guo et al. (2019);
			Scavenges ROS	Lee et al. (2018)
			Located on the cell wall of invading hyphal cells	
	*ECM33*	GPI-anchored protein	Cell wall morphology	Fernando et al. (2019)
	*PP2A*	Phosphatase	Essential	Yu et al. (2014)
	*STE7*	kinase	Vegetative growth	Gu et al. (2015)
			Conidiation	
			Mycotoxin production	
			Penetration	
	*PHO8*	Phosphatase	Essential	Yun et al. (2015)
	*CNA1*	Phosphatase	Essential	Yun et al. (2015)
	*Fg07304*	Phosphatase	Essential	Yun et al. (2015)
	*GIC1*	Phosphatase	Conidiation	Yun et al. (2015)
	*PSR2*	Phosphatase	Vegetative growth	Yun et al. (2015)
			Pigmentation	
			Conidia morphology	
	*ARC15*	Phosphatase	Only defects in virulence	Yun et al. (2015)
	*GLX*	glyoxal oxidase	Mycotoxin production	Song et al. (2016)
			localized to cell membrane	
	*ARB1*	ATP-binding protein	Infective growth	Yin et al. (2018a)
			Oxidative stresses	
			Cell wall integrity	
			Mycotoxin production	
			Sexual and asexual development	
	*SKP1*	E3 ubiquitin ligases	Essential	Chen et al. (2023)
	*LIP50, TAG*	Lipases	Only defects in virulence	Kim et al. (2023); Zhang et al. (2016)
	*PCT1*	Cytidylyltransferases	Essential	Wang et al. (2019)
	*FGSG_10825*	Mating-type	Sexual development	Kim et al. (2015a)
	*OB031, HEX1*	TF	Only defects in virulence	Son et al. (2011)
	*CMK1*	Kinase	Ascospore formation	Wang et al. (2011)
	*ALDH (FGSG_02273)*	Aldehyde Dehydrogenase	Essential	Tang et al. (2023)
	*ALDH (FGSG_04194)*	Aldehyde Dehydrogenase	Regulating acetaldehyde dehydrogenase activity	Tang et al. (2023)
			Accumulation of lipid droplets	
			Conidial germination	
	*CWM1*	Cell wall mannoprotein	Vegetative growth	Zhang et al. (2019a)
			Cell wall integrity	
*L. theobromae*	*LtScp1*	Effectors, a LysM-containing protein	(Site directed mutagenesis)	Peng et al. (2022)
			Interacted with chitinase VvChi4	
*M. oryzae*	*CNX1*	Calnexin	Mycelial growth	Chen et al. (2020)
			Conidiation	
			Invasive hyphal growth	
	*GTB1*	Glucosidase II β-subunit Gas2	Mycelial growth	Chen et al. (2020)
			Conidiation	
			Invasive hyphal growth	
	*SLP1*	Secreted LysM domain protein	(Site directed mutagenesis)	Chen et al. (2014); Jones et al. (2021)
			Sequesters chitin oligosaccharides	
			Apoplastic effectors	
	*BAS4*	Biotrophy-associated secreted protein 4	Apoplastic effectors	Chen et al. (2014); Jones et al. (2021)
	*GPI7*	Glycosylphosphatidylinositol	Penetration and invasive growth	Liu et al. (2020)
			Cell wall biogenesis	
*P. capsici*	*Pcipg2*	Polygalacturonase	Studied only for virulence	Sun et al. (2009)
*P. sojae*	*GPI16*	GPI transamidase component protein	(Site directed mutagenesis)	Zhang et al. (2021)
			Cyst germination	
			Oospore production	
*A. fumigatus*	*GP56*	Major antigen/allergen with a molecular weight of 56 kD	Studied only for virulence	Nigam et al. (2003)
*C. albicans*	*CaHWP1*	Hyphal wall protein 1	Positive regulators of hyphal development	Sharkey et al. (1999); Staab et al. (2013)
	*CaALS1*	Agglutinin-like sequence	Studied only for virulence	Hoyer et al. (2008); Kamai et al. (2002)
		cell-surface glycoproteins		
	*CaALS3*	Agglutinin-like sequence	Studied only for virulence	Hoyer et al. (2008); Zhao et al. (2004)
		cell-surface glycoproteins		
	*CaALS2*	Agglutinin-like sequence	Studied only for virulence	Hoyer et al. (2008); Zhao et al. (2005)
		cell-surface glycoproteins		
	*CaMP65*	Putative β-glucanase mannoprotein	Hyphal morphogenesis	Sandini et al. (2007)
			Fungal cell adherence	
*C. neoformans*	*PLB1*	Glycoproteins phospholipase	Intracellular infection	Evans et al. (2015); Thak et al. (2020)
			Cell body morphology	
	*MP65*	Immunodominant 65-kDa-MP antigen	Studied only for virulence	Pietrella et al. (2002)
	*CIG1*	Extracellular mannoprotein	Iron acquisition	Cadieux et al. (2013)
			Growth on heme	
	*MP84*	Mannoprotein	Not studied	Teixeira et al. (2014)
	*CMP1*	Cryptococcus mannoprotein 1	Capsule formation	Han et al. (2020)
			Localized in the vacuole	
